# Integrative genome-wide analysis reveals EIF3A as a key downstream regulator of translational repressor protein Musashi 2 (MSI2)

**DOI:** 10.1093/narcan/zcac015

**Published:** 2022-05-02

**Authors:** Shilpita Karmakar, Oscar Ramirez, Kiran V Paul, Abhishek K Gupta, Vandana Kumari, Valentina Botti, Igor Ruiz de los Mozos, Nils Neuenkirchen, Robert J Ross, John Karanicolas, Karla M Neugebauer, Manoj M Pillai

**Affiliations:** Section of Hematology, Yale Cancer Center, New Haven, CT 06511, USA; Section of Hematology, Yale Cancer Center, New Haven, CT 06511, USA; Section of Hematology, Yale Cancer Center, New Haven, CT 06511, USA; Section of Hematology, Yale Cancer Center, New Haven, CT 06511, USA; Section of Hematology, Yale Cancer Center, New Haven, CT 06511, USA; Department of Molecular Biophysics and Biochemistry, Yale University School of Medicine, New Haven, CT 06510, USA; Institute of Neurology, University College London and The Francis Crick Institute, London NW1 1AT, UK; Department of Cell Biology, Yale University School of Medicine, New Haven, CT 06511, USA; Department of Cell Biology, Yale University School of Medicine, New Haven, CT 06511, USA; Program in Molecular Therapeutics, Fox Chase Cancer Center, Philadelphia, PA 19111, USA; Department of Molecular Biophysics and Biochemistry, Yale University School of Medicine, New Haven, CT 06510, USA; Section of Hematology, Yale Cancer Center, New Haven, CT 06511, USA; Department of Pathology, Yale University School of Medicine, New Haven, CT 06511, USA; Yale Stem Cell Center, Yale University School of Medicine, New Haven, CT 06510, USA

## Abstract

Musashi 2 (MSI2) is an RNA binding protein (RBP) that regulates asymmetric cell division and cell fate decisions in normal and cancer stem cells. MSI2 appears to repress translation by binding to 3′ untranslated regions (3′UTRs) of mRNA, but the identity of functional targets remains unknown. Here, we used individual nucleotide resolution cross-linking and immunoprecipitation (iCLIP) to identify direct RNA binding partners of MSI2 and integrated these data with polysome profiling to obtain insights into MSI2 function. iCLIP revealed specific MSI2 binding to thousands of mRNAs largely in 3′UTRs, but translational differences were restricted to a small fraction of these transcripts, indicating that MSI2 regulation is not triggered by simple binding. Instead, the functional targets identified here were bound at higher density and contain more ‘UAG’ motifs compared to targets bound nonproductively. To further distinguish direct and indirect targets, MSI2 was acutely depleted. Surprisingly, only 50 transcripts were found to undergo translational induction on acute loss. Using complementary approaches, we determined eukaryotic translation initiation factor 3A (EIF3A) to be an immediate, direct target. We propose that MSI2 downregulation of EIF3A amplifies these effects on translation. Our results also underscore the challenges in defining functional targets of RBPs since mere binding does not imply a discernible functional interaction.

## Graphical Abstract

**Graphical Abstract:**
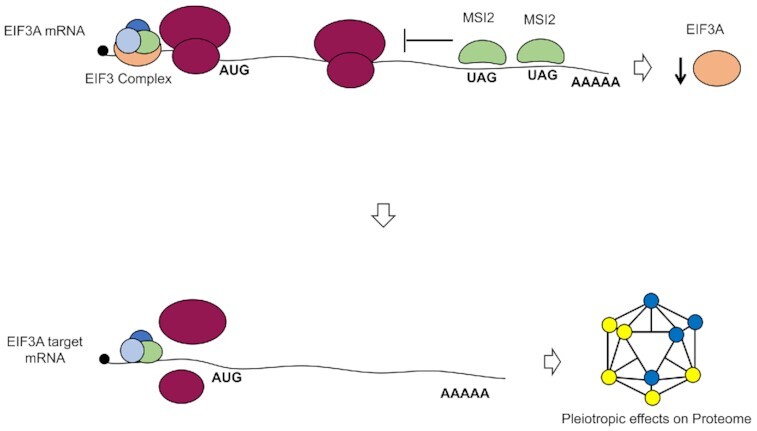
Musashi 2 regulates expression of eukaryotic translation initiation factor 3A, which in turn causes pleiotropic changes to the proteome.

## INTRODUCTION

RNA binding proteins (RBPs) encompass a diverse group of proteins that regulate all aspects of RNA biology. The Musashi proteins (MSI1 and its homologue MSI2) are highly conserved across metazoans and contain two distinct RNA recognition motifs (1). MSI proteins are thought to bind to the 3′ untranslated region (3′UTR) of specific transcripts and regulate their translation. Of the two MSI homologues, MSI1 is expressed primarily in neurons (2). In contrast, MSI2 is ubiquitous, but with high levels in some tissues such as hematopoietic stem and progenitor cells, where its downregulation coincides with stem cell differentiation (3). A role for dysregulated MSI2 expression in cancer was first reported in aggressive myeloid neoplasms such as chronic myelogenous leukemia (CML) in myeloid blast crisis and aggressive acute myeloid leukemia (3,4). High expression and critical regulatory roles have since been reported in other malignancies such as colorectal cancer (5), pancreatic cancer (6), medulloblastoma (7), breast cancer (8,9), lung cancer (10–12) and chronic lymphocytic leukemia (13).

The critical role of MSI proteins in regulating asymmetric cell division and cell fate suggests a mechanism that regulates specific molecular targets. A number of genome-wide approaches have been utilized to define the RNA interactome of MSI1 and MSI2. These include CLIP-seq (or HITS-CLIP) in mouse keratinocytes, leukemic cell lines, embryonic kidney cell lines and intestinal epithelium (14–18). Additional complementary techniques such as SELEX (systematic evolution of ligands by exponential enrichment) and TRIBE (targets of RNA binding proteins identified by editing) have also been implemented for this purpose (19,20). These studies demonstrated that MSI proteins bind to thousands of transcripts in a cell context-specific manner. While many functionally relevant targets were noted to be bound by MSI homologues in each of these studies, simultaneous analysis of functional regulation of these bound targets was not performed. The impact of these studies is limited by a lack of knowledge about which targets are directly bound by Musashi proteins and, in the case of possible indirect targets, what critical molecular pathways Musashi regulates to yield the observed changes in gene expression when overexpressed or knocked down.

In this study, we sought to answer this question: Which of these bound targets are translationally modulated by MSI proteins? We hypothesized that MSI2 affects the translation of only a subset of the transcripts it binds to. To test this, we integrated two genome-wide approaches—individual nucleotide resolution cross-linking and immunoprecipitation (iCLIP) and polysome profiling—to address the relationship between MSI2 binding and translational regulation. Because we are interested in understanding the role of MSI2 in cancers where MSI2 is expressed, we established FLAG-tagged MSI2 expressed in K562 cells as a model system. The K562 cell line was derived from the blast crisis stage of CML patients and has high constitutive expression of MSI2 (14,21). Analysis of the data reveals that although MSI2 binds to the 3′UTR of >4000 transcripts in this study, only a fraction (2.6%) of these have changes detected through polysome profiling. Through acute depletion of MSI2 and polysome profiling, we also identify eukaryotic translation initiation factor 3A (EIF3A) as a critical downstream effector of MSI2. Additionally, our results argue for the need to incorporate functional assays in tandem with CLIP-seq approaches to distinguish binding and regulatory functions of an RBP.

## MATERIALS AND METHODS

### Cell culture

Cell lines were obtained from the American Type Culture Collection. K562 cells (21) and derivatives were cultured in RPMI 1640 supplemented with 10% fetal bovine serum (FBS). HEK293T and NIH3T3 cells were grown in Dulbecco’s modified Eagle medium supplemented with 10% FBS. Puromycin selection (for stable FLAG-MSI2 overexpression or shRNA-mediated knockdown) was performed at 1 μg/ml concentration. Neomycin (G418) selection (for inducible shRNA clones) was performed at 800 μg/ml concentration. Single-cell clones were selected after 10–14 days of selection by plating cells in methylcellulose as previously described (22).

### Cloning, plasmid constructs and viral vector production

The human MSI2 open reading frame (NM_138962.2) was PCR amplified from complementary DNA (cDNA) and cloned into the BamHI and EcoRI sites of the pBABE-puro retroviral vector with an N-terminal FLAG tag. Stable lentiviral vectors expressing shRNA targeting MSI2 and control shRNAs were obtained from Sigma-Aldrich (Mission lentiviral system, based on the pLKO.1 vector; clone details are provided in the Supplementary Methods) and confirmed for their knockdown activity by RT-PCR and western blotting. Lentiviral vector pLKO-Tet-On (Addgene plasmid #21916) was used to generate inducible knockdown clones of MSI2 and EIF3A in the K562 cell line (23) (details are provided in the Supplementary Methods). To create the pMS2-LUC-3′UTR vector, Renilla luciferase was cloned from psiCHECK2 into pcDNA3.1(+) followed by 3′UTR and five stem loops of MS2 [amplified from pSL-MS2-6X (Addgene #27118)]. MS2 coat binding protein (MS2-BP) was amplified from pSL-MS2-6X (Addgene #27118), and an N-terminal FLAG was added and cloned in place of neomycin resistance cassette. The vector scheme is shown in Figure 7A (details of the cloning scheme and a vector map are provided in the Supplementary Methods). Retroviral and lentiviral vectors were produced by the co-transfection of respective proviral plasmids with appropriate helper and envelope plasmids and transduced into K562 cells as previously described (22).

### iCLIP for MSI2

iCLIP for FLAG-MSI2 was performed as previously reported (24) with minor variations (25). Three single-cell clones of K562 cells expressing FLAG-tagged MSI2 were isolated and verified for stable expression of FLAG-MSI2 (Supplementary Figure S1A). Forty million K562 cells expressing FLAG-MSI2 were cross-linked twice (4 and 2 mJ pulses using UV Stratalinker 2400, Stratagene) and stored at −80°C prior to analysis. FLAG-MSI2 cross-linking to RNA by UV was confirmed (Supplementary Figure S1B) and RNase A digestion was optimized to attain the optimal distribution of the MSI2-RNA smear [above the predicted molecular weight of FLAG-MSI2 (37 kDa); Supplementary Figure S1C]. RNA-seq libraries were prepared from RNA isolated from corresponding batches of cells. Illumina-compatible libraries were prepared from the isolated RNA (see the ‘Materials and Methods’ section and Supplementary Figure S1C) and sequenced to a depth of ∼50 million single-end reads per sample. PCR duplicates were eliminated by introducing 5 bp random sequences during the reverse transcription step as unique molecular identifiers. After mapping to the human genome (hg19), cross-link sites and clusters were determined by the iCount algorithm (26).

### Polysome profiling

Polysome profiling was performed as previously reported (27,28) using single-cell clones of K562. Briefly, ∼40 million cells in log-phase growth were treated with cycloheximide (1 μg/ml) for 10 min, lysed in TMK lysis buffer, cleared of debris by centrifugation and loaded on a 10–60% sucrose gradient. Polysome fractionation was achieved by ultracentrifugation and individual fractions were collected (46 fractions of ∼800 μl) using the Teldyne ISCO automated fraction collector with continuous monitoring of the absorbance at 254 nm. Fractions corresponding to heavier polysomes were pooled. Total and polysomal RNAs were isolated by Trizol (Life Technologies, Carlsbad, CA). RNA-seq libraries for polysomal RNA and total RNA were prepared using the Illumina TruSeq Kit and were sequenced on the Illumina HiSeq2000 (single-end, 50 bp). A detailed protocol for polysome profiling is provided in the Supplementary Methods.

### Reverse transcription and quantitative PCR

cDNA was prepared from ∼0.5 μg total RNA [isolated using RNeasy Mini Kit (QIAGEN)] using M-MuLV reverse transcriptase (New England Biolabs) as per manufacturer’s instructions. Genomic DNA contamination was eliminated using on-column DNase digestion and RNA integrity confirmed by a bioanalyzer. Oligonucleotides for quantitative PCR (qPCR) targets were selected using the Primer-BLAST tool (https://www.ncbi.nlm.nih.gov/tools/primer-blast/), which uses the BLAST algorithm and predicts specificity of primer pairs. Primers were ordered from IDT and sequences are provided in the Supplementary Methods. qPCR of this reverse transcription reaction was performed using KAPA SYBR FAST One-Step qRT-PCR Master Mix (2×) (KAPA Biosciences) with ∼20 ng of cDNA on a CFX Touch Real-Time PCR Detection System (Bio-Rad) using manufacturer’s instructions. The following conditions were used: denature for 2 min at 95°C, followed by 40 cycles of 95°C for 5 s and 60°C for 30 s. The melting curve was determined by stepwise denaturation (0.5°C increments to 95°C). Quantitation was performed using the 2^−^^ΔΔCT^ method with a housekeeping control gene (GAPDH) (29). All primer pairs were validated to have sharp melting curves and correct amplicon size. Control reactions with no templates were performed during each experiment to confirm that no amplicon was formed.

### Luciferase assay

The 3′UTR of EIF3A (or mutants lacking three consecutive UAG motifs) was cloned downstream of the Renilla luciferase in the XhoI and NotI sites of the psiCHECK2 vector (Promega, Madison, WI; sequence details are provided in the Supplementary Methods). Forty nanograms of the psiCHECK2 plasmid was transiently co-transfected with 320 ng of MSI2-pcDNA3.1(+) in NIH3T3 (30) cells using TransIT-X2 (Mirus Bio) transfection reagent following manufacturer’s instructions and luciferase activity (Renilla and firefly) was measured 24 h later with the Dual Glow-Stop and Glow luciferase kit (Promega) using a BioTek luminometer (Synergy).

### Western blot analysis

For western blot analysis, cells were lysed using 1× RIPA buffer [10 mM Tris–HCl (pH 8.0), 1 mM EDTA, 0.5 mM EGTA, 1% Triton X-100, 0.1% sodium deoxycholate] supplemented with 1× Complete Mini EDTA Protease Inhibitor Cocktail (Roche), incubated on ice for 15 min, and the supernatant was isolated by centrifugation. Protein concentration was determined using the DC protein assay (Bio-Rad) following the manufacturer’s recommendations. Thirty micrograms of total protein was resolved on 10% precast SDS-PAGE gel (Bio-Rad) and transferred to a methanol-preconditioned PVDF membrane by wet transfer for 90 min at 100 V. Membranes were blocked with 5% non-fat dry milk and probed with the appropriate primary and secondary antibodies. Immunoreactive bands were visualized using electrochemiluminescence (Roche). Details of antibodies used and other conditions for blotting are summarized in the Supplementary Methods.

### FLAG-MS2-BP pull down to demonstrate direct binding of MSI2 to putative targets

HEK293 cells (with a high baseline MSI2 expression) at 70–80% confluence in a 10-cm dish were transfected with 10 μg of each of the pMS2-LUC-3′UTR with TransIT-X2 (Mirus Bio) according to manufacturer’s instructions. Forty-eight hours later, cells were harvested, lysed in NET2 buffer and protease inhibitor cocktail (Roche), sonicated on ice, centrifuged to remove cellular debris and immunoprecipitated for the FLAG tag using anti-flag-M2 agarose beads (Sigma). Bound protein was eluted with 200 μg/ml of 3× FLAG peptide (Sigma). Input and immunoprecipitation (IP) were analyzed by immunoblotting (against MSI2 and β-actin) as detailed earlier. Further details are provided in the Supplementary Methods.

### Metabolic labeling for nascent protein determination

Ten million cells were washed and grown in methionine-free media for 30 min to deplete methionine stores and then supplemented by l-azidohomoalanine (l-AHA) (50 μM final concentration for 4 h). Cell lysates were measured for OD at 260 and equal protein amounts were subjected to click chemistry using biotin alkyne (Click Chemistry Tools) as per manufacturer’s instructions. Total protein was precipitated with trichloroacetic acid, suspended in suspension buffer [8 M urea in 50 mM Tris–HCl (pH 7.4), 1 mM DTT] and biotin-tagged protein was isolated using streptavidin MyOne C1 magnetic beads (Thermo Fisher). Beads were washed three times with 8 M urea in Tris–HCl (pH 7.4), boiled for 10 min in SDS buffer and supernatant was subjected to western blotting.

### Bioinformatic analysis and statistics

Details of the bioinformatic analysis are provided in the Supplementary Methods. The Mann–Whitney nonparametric testing was used to determine statistical significance for comparisons of next-generation sequencing datasets. Overlap between datasets was performed using Fisher’s exact test (‘Gene overlap’ function of R package Bioconductor). Student’s *t*-test was used for other comparisons.

## RESULTS

### Thousands of transcripts are bound by MSI2 in K562 cells

**Figure 1.**
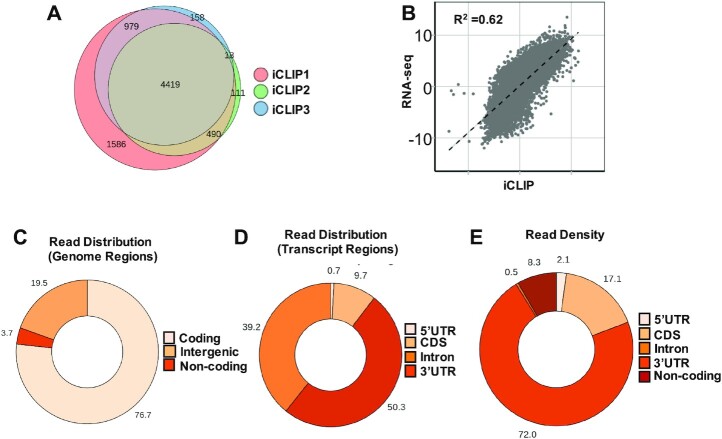
iCLIP analysis of FLAG-MSI2 across genomic and transcriptomic regions. (**A**) Overlap of transcripts with 3′UTR peaks between three replicate iCLIP experiments. Transcripts with high-confidence cross-link clusters determined by iCount were used for analysis. Four thousand four hundred nineteen transcripts were found to have cross-link clusters in 3′UTRs in all three replicates. (**B**) Scatter plot showing correlation between MSI2 binding (normalized FPKM from three iCLIP experiments) and transcript abundance (normalized RNA-seq FPKM), *R*^2^ = 0.6245. (**C**) Distribution of aligned iCLIP clusters across genomic regions (protein-coding genes, noncoding genes and intergenic regions). Pie chart plotted as a percentage for each region (total 100%). (**D**) Distribution of iCLIP cross-link clusters across different transcript regions (5′UTR, CDS, 3′UTR and introns). Pie chart plotted as a percentage for each region (total 100%). (**E**) Distribution of absolute read counts normalized to the length of each transcript region. Total number of reads that were uniquely aligned to each of the regions was determined (FPKM). This was normalized to the total length of those regions. Pie chart plotted as a percentage for each region (total 100%).

Like other protocols that detect RNA–protein interactions, iCLIP utilizes UV radiation to cross-link RNA to adjacent protein moieties at 0 Å, allowing stringent washing during IP of RNA–protein complexes (31). RNA cross-linking to FLAG-MSI2 was confirmed and RNase conditions were optimized prior to library preparation (Supplementary Figure S1A–D). In iCLIP, cDNA stop sites are annotated as cross-link sites, and regions with significant clustering of cross-link sites are designated as ‘cross-link clusters’ (31). Cross-link clusters that met our statistical cutoff [false discovery rate (FDR) < 0.05] and were represented in at least two of three biological replicates were designated as high-confidence clusters and used for further analysis. A high degree of overlap was found between the target genes identified between the three biological replicates (Figure 1A). The positive correlation (*R*^2^ = 0.625) between transcript abundance and iCLIP abundance (Figure 1B) is typical of iCLIP experiments, given that RNAs must be expressed to be detected; the distribution confirms that a broad range of expression levels are represented in the iCLIP dataset.

Most cross-link clusters (76.7%) were identified in protein-coding genes, with noncoding genes and intergenic regions encompassing only 3.7% and 19.5% of clusters, respectively (Figure 1C). Within protein-coding genes, clusters were enriched in 3′UTR (50.3%), in agreement with the purported role of MSI2 as a 3′UTR binding protein (Figure 1D). Only 0.7% of clusters were localized to 5′UTR, and 9.7% were in protein-coding regions (CDS). 39.2% of clusters were noted to be localized to introns. The density of these clusters (normalized to the respective length of the transcript region) is shown in Figure 1E. Such normalized density was by far the highest in 3′UTR (72%) followed by CDS (17.1%). 8.3% of the density belonged to 5′UTR and 2.1% to the noncoding region. Introns had the lowest density (0.5%) despite having highest total reads aligning to it. The large proportion of total iCLIP reads aligning to introns was unexpected, given that MSI proteins are thought to be cytoplasmic in location due to their primary role in translational repression. However, recent reports have suggested a nuclear localization for MSI2 during specific phases of the cell cycle (32) and associated with neurodegenerative disorders (33). Re-association of RBPs with target RNA after cell lysis has been reported (34), which may represent another source of intronic reads. Intronic reads have also been reported for MSI1 CLIP-seq (35). Taken together, our results suggest predominant binding of MSI2 to the 3′UTR region of protein-coding transcripts, with sparse binding to other regions, including introns.

### MSI2 binding motifs are enriched for the UAG motif and polyU motifs, but not at translation stop sites

**Figure 2.**
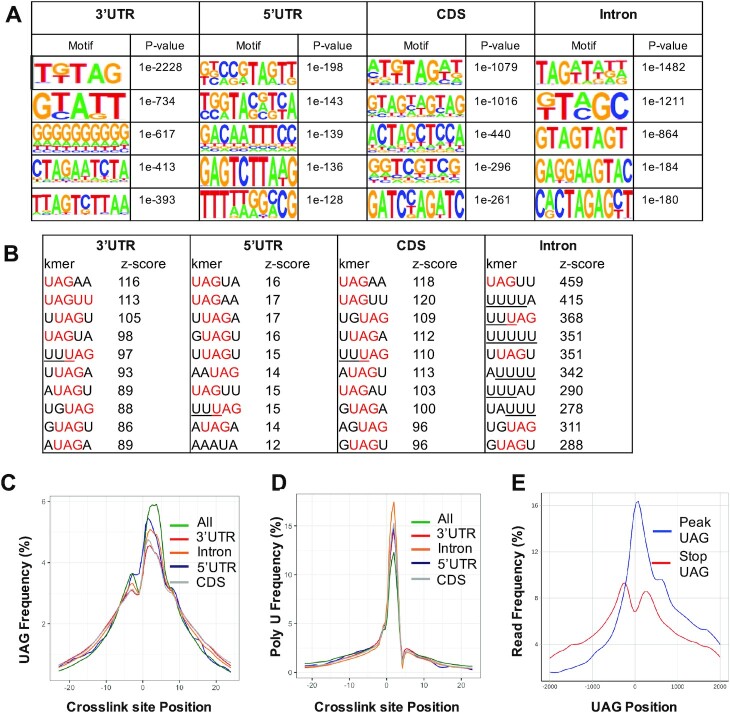
Sequence motifs in FLAG-MSI2 iCLIP datasets. (**A**) Motifs identified in regions −20 to +20 from high-confidence iCLIP cross-link sites (FDR < 0.05) by the HOMER algorithm. Top 5 motifs along with the corresponding *P*-values are shown for each of the transcript regions (5′UTR, CDS, 3′UTR and introns). (**B**) Ten most frequent 5-mers identified in a window of −20 to +20 nucleotides with respect to cross-link sites for each of the transcript regions (5′UTR, CDS, 3′UTR and introns). UAG motifs within these 5-mers are denoted in red and poly-U stretches (>3) are underlined. (**C**) Distribution of ‘UAG’ motifs upstream and downstream (−25 to +25 nucleotides) of the cross-link sites. Frequency distribution across each transcriptomic region (5′UTR, CDS, 3′UTR and introns) along with aggregate distribution across all regions (‘All’) is shown. Enrichment of UAG motif is seen around cross-link sites. (**D**) Distribution of polyU motifs (three or more) with respect to cross-link site positions, plotted in a similar fashion to that in panel (C). Enrichment for poly-T stretches is seen around the cross-link sites. (**E**) Distribution of read density at and around (2 kb up- and downstream) UAG within iCLIP peaks. Red lines indicate UAG sites that are annotated translation stop sites and blue lines denote UAG within iCLIP peaks, and are not denoted to be stop sites.

Previous studies using genome-wide profiling of MSI2-bound RNA or SELEX have shown that MSI proteins bind to UAG in RNA (19,36,37) or contain polyU stretches (16,35). We implemented two strategies to search for motif enrichment in the iCLIP datasets. We first examined the enrichment of motifs in a window from −20 to +20 bp from the high-confidence cross-link sites using the HOMER algorithm (38). Motifs thus enriched typically included ‘UAG’-containing motifs across the different transcriptomic regions (Figure 2A). We then examined enrichment of specific pentamers in the cross-link clusters, as determined by iCount (31). Notably, these pentamers revealed enrichment for UAG motifs or high U content (Figure 2B). Finally, we determined the distribution of the UAG motif and U-rich sequences from high-confidence cross-link sites (Figure 2C and D, respectively). These motif features were found to be enriched around the cross-link sites. Combined, our results confirm an enrichment of UAG- or polyU-containing motifs in MSI2 binding sites. A modest bias toward uracil-containing stretches is characteristic of CLIP dataset due to preferential cross-linking (39), which may explain enrichment for polyU motifs.

Given that UAG is a termination codon, we also asked whether there was a predilection for increased MSI2 binding at annotated UAG stop codons. We compared read density at UAG sites across the iCLIP peak regions as well as UAG codons in the same genes (Figure 2E). Decreased relative density at annotated stop UAG compared to those in peaks suggests that enrichment of UAG motifs is not dependent on their function as stop codons.

### Polysome profiling reveals distinct effects on translatome compared with transcriptome

**Figure 3.**
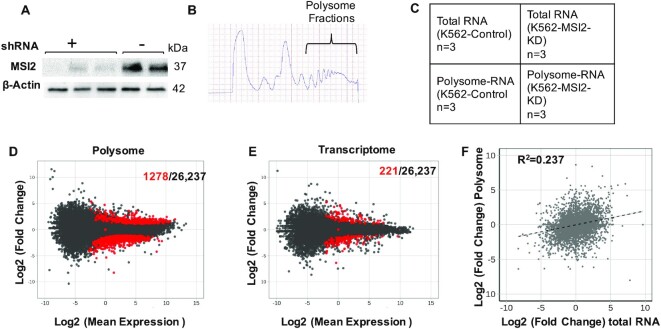
Polysome analysis of MSI2-KD and control cells. (**A**) Western blot of MSI2-KD and control K562 cells showing loss of MSI2 expression by stable expression of anti-MSI2 shRNA. (**B**) Absorbance at 254 nm of sucrose gradients for polysome isolation. Higher density fractions as indicated were pooled for RNA isolation. (**C**) Experimental scheme for polysome analysis. RNA-seq was performed on paired polysomal RNA and total RNA from wild-type and MSI2-KD cells (in three biological replicates). (**D**) MA plot (log_2_ mean expression plotted against log_2_ mean expression) for polysome profiling (MSI2 knockdown versus control). Red dots represent 1278 transcripts that changed significantly among the 26,237 total transcripts (black dots) as defined by *q* value of <0.05. (**E**) MA plot for total cellular RNA, plotted similarly to that in panel (D). Red dots represent 221 transcripts that change significantly among the 26,237 total transcripts (in black) as defined by *q* value of <0.05. (**F**) Scatter plot of changes in transcript abundance, polysome versus total RNA (log_2_FC). *R*^2^ of dispersion was calculated to be 0.237.

Specific binding of MSI2 to thousands of targets as revealed by iCLIP was surprising, given the specific biological roles of MSI2 in cell fate decisions. We hypothesized that only a subset of target transcripts bound by MSI2 actually undergo functionally relevant translational regulation, and thus be defined as translational targets of ‘productive’ MSI2 binding. To define this subset, we first generated stable MSI2 knockdown (MSI2-KD) and control cells with lentiviral shRNA constructs. After verifying reduction of MSI2 (Figure 3A and Supplementary Figure S2), polysome profiling was performed (27). Polyribosomes or polysomes are aggregates of two or more ribosomes assembled on mRNA undergoing efficient translation (40). By comparing the change in abundance of transcripts associated with polysomes to the change in total transcript levels, changes in translation can be inferred (41). Polysome and transcriptome profiles were generated for each of these clones in three replicates (Figure 3B and C). Potential productive translational targets of MSI2 were identified as those transcripts that changed at least 2-fold by polysome profiling without significant changes in total cellular RNA, as described previously (42). By this criterion, a total of 1278 high-confidence genes were identified (Figure 3D and Supplementary File S1). In contrast, only 221 genes changed by total RNA levels (Figure 3E and Supplementary File S1). Concomitantly, transcripts with altered transcript abundance in polysome versus total RNA fractions were poorly correlated (*R*^2^ = 0.237, Figure 3F). In all, 2.3% of genes underwent translational change (defined as altered polysome-specific mRNA abundance), while only 0.39% changed transcriptionally. Together, our results suggest a distinct role for MSI2 in translational regulation.

### Integration of iCLIP and polysome profiling identifies high-confidence targets of MSI2

**Figure 4.**
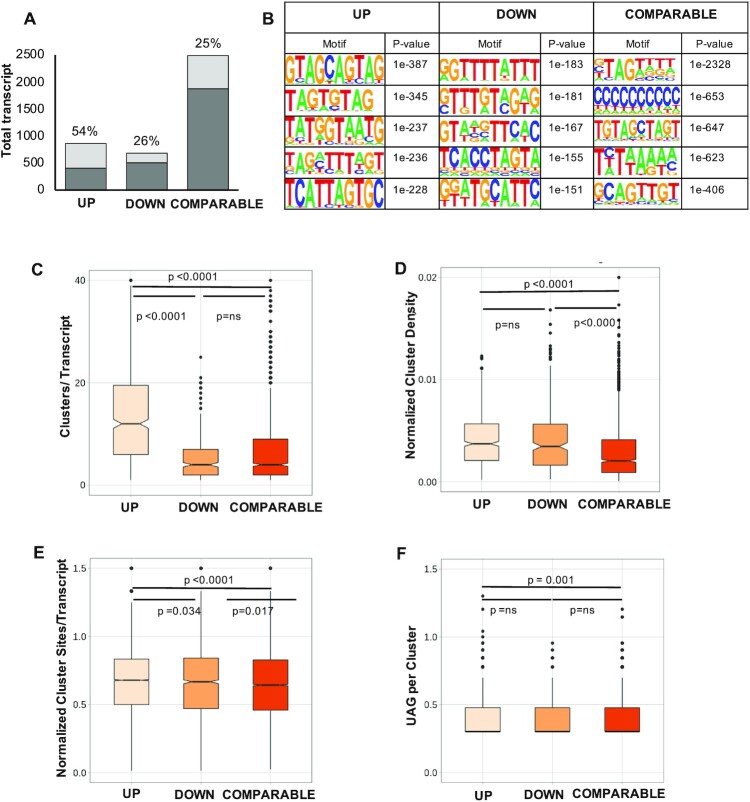
Features of transcripts translationally changing upon MSI2 knockdown and having high-confidence iCLIP peaks in 3′UTR. (**A**) Proportion of genes in the polysome-regulated groups that have iCLIP peaks within the transcript regions. Four hundred fifty-eight of the 851 (54%) upregulated genes (UP) have iCLIP peaks, while only 178/673 (26%) of downregulated genes (DOWN) and 617/2488 (25%) of unchanged genes (COMPARABLE) have similar iCLIP peaks. Transcripts with a minimum mean FPKM of 10 in either of MSI2-KD or control polysome groups were included in the analysis. The light gray shading represents the proportion of altered genes from each group that have iCLIP peaks in the transcript; dark gray shading represents the remaining genes in each group. (**B**) Motifs identified in regions −10 to +10 bp of cross-link sites as determined by the HOMER algorithm in the upregulated, downregulated or comparable groups. Top 5 motifs along with the corresponding *P*-values are shown for each of the transcript regions (5′UTR, CDS, 3′UTR and introns). (**C**) Total number of cross-link clusters per transcript among those transcripts with iCLIP clusters. Results are segregated in three groups based on polysome profiling results. Box plots within violin plots show mean value as well as *P*-value determined by the Mann–Whitney test. (**D**) Density of cross-link clusters (total number of high-confidence clusters normalized to the length of the transcript) for each of the three groups, plotted similarly to that in panel (C). (**E**) Density of cross-link sites (total number of cross-link sites with FDR < 0.05 that fall within the transcript coordinates normalized to length of the transcript) for each of the three groups, plotted similarly to that in panel (C). (**F**) Total number of UAG motifs (log_2_) found within high-confidence cross-link clusters for each of the three groups. The line in the middle of violin plot denotes mean value.

Since polysome profiling was performed in cells with sustained MSI2 knockdown, it is not evident which of the transcripts are direct targets of MSI2 and which are indirectly regulated (through other downstream mediators). To determine direct MSI2 targets, we first cross-referenced our list of MSI2-dependent, translationally regulated target transcripts with mRNAs with high-confidence 3′UTR iCLIP cross-link clusters (Supplementary File S2). While 53.8% genes that were translationally upregulated in response to MSI2 knockdown had iCLIP clusters within their 3′UTR, only 26.5% of those downregulated by MSI2 knockdown had similar 3′UTR peaks. A similar proportion (24.8%) of transcripts with no change in translation (COMPARABLE) also had peaks in their 3′UTR (Figure 4A). We next sought to determine features that distinguish productive MSI2 targets from nonproductive binding events. Three groups of MSI-bound transcripts were identified for detailed analysis: (i) those translationally upregulated in response to MSI2 knockdown with iCLIP peaks in 3′UTR (UP); (ii) those translationally downregulated by MSI2 knockdown with similar iCLIP peaks (DOWN); and (iii) mRNAs translationally unchanged with iCLIP peaks (COMPARABLE). We analyzed several attributes of genes within these subsets to determine what distinguishing features might predict productive binding events, including primary sequence motifs, density of cross-link clusters, density of motifs and secondary structure constraints. Primary sequence motif analysis in these three subsets showed an enrichment for UAG-containing sequences in the UP dataset (Figure 4B). We next looked at the possibility that productive binding by MSI2 requires multiple molecules binding to the target, which could be inferred from the number and density of cross-link clusters in the iCLIP (31) datasets (cross-link clusters are those regions within iCLIP alignments that cluster together) (31). UP targets were found to have a significantly higher number of total cross-link clusters per transcript and density of cross-link clusters normalized to transcript length (Figure 4C and D, respectively). Additionally, the UP targets also had higher number of individual iCLIP cross-link sites with higher UAG motif (Figure 4E and F). Together, our results show that productive binding of MSI2 to downregulate translation is correlated with high-density binding of MSI2. The wide distribution of values in our analysis (Figure 4C–F) suggests that productive binding may have additional requirements (such as binding by other RBPs). Given that secondary structure of target transcripts is now known to be a major determinant of RBP–RNA interactions (43), we analyzed the secondary structure of RNA around cross-link sites in the three groups of MSI-bound transcripts using the CapR algorithm (44). We found that cross-link sites or clusters from the three subgroups did not differ from each other with regard to their likelihood to form secondary structures or their relative accessibility (Supplementary Figure S3A–F).

### Numerous cancer-relevant genes and pathways change upon loss of MSI2

**Figure 5.**
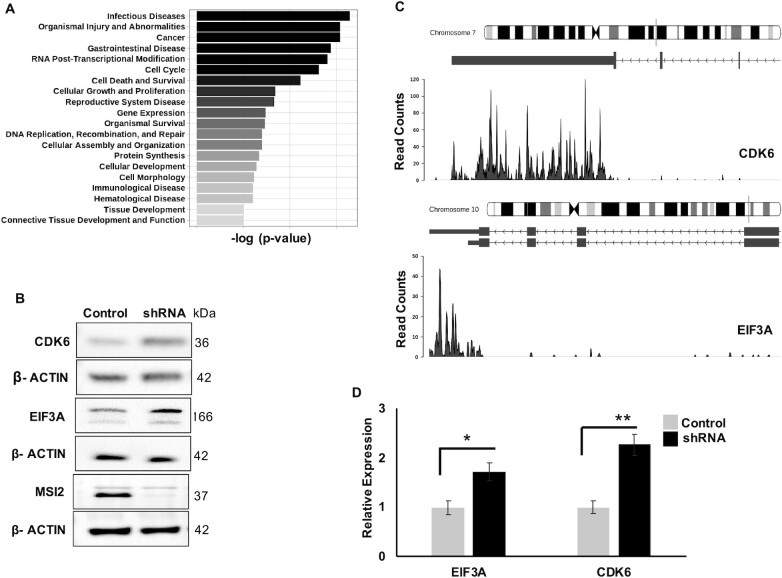
Biological pathways and genes targeted by MSI2. (**A**) Pathway analysis of MSI2-regulated genes for diseases and functions. The analysis was conducted with high-confidence genes showing differential regulation in genes upregulated in the polysome upon MSI2 knockdown. Plotted are the top 20 pathways and corresponding –log_10_(*P*-value). (**B**) Western blot for candidate genes CDK6 and EIF3A upon stable lentiviral expression of either control (scramble) shRNA or MSI2 shRNA. Also included are western blots for MSI2 (showing knockdown of MSI2 with shRNA expression) and loading control (β-actin). (**C**) Genome coverage plots of CDK6 and EIF3A corresponding to 3′UTR from iCLIP for MSI2. (**D**) Quantification of EIF3A and CDK6 western blots (compared with loading control) from three replicates (β-actin). * denotes a *P*-value of 0.033 and ** denote a *P*-value of 0.008. EIF3A changed by 1.61-fold and CDK6 changed by 1.72-fold from all the replicate gels with β-actin as loading control.

To determine global changes brought about by MSI2 depletion, we performed pathway analysis of transcripts that changed at the level of translation using the ingenuity pathway analysis algorithm. Transcripts found translationally upregulated upon MSI2 knockdown were highly enriched within categories of cancer, cell cycle, cell death and differentiation (Figure 5A). We then analyzed individual transcripts predicted to be upregulated upon MSI2 knockdown without discernible change in total mRNA levels. These included several encoding cancer-relevant proteins, including EIF3A, MYC, CDK6, SP1, RAD21, USP28, FOXO family proteins and STAT signaling regulators (full list is provided in Supplementary File S1). To determine whether the transcripts changing in polysome profiling were also changing at their protein expression levels, we performed western blot and densitometric analyses by normalizing with β-actin loading control (Figure 5B–D). Protein levels of EIF3A and CDK6 showed 1.61 and 1.72 fold increase as predicted from polysome data. Importantly, these transcripts also had putative binding sites in their respective 3′UTR as shown by iCLIP (Figure 5C). Some of the targets (such as SP1, C-MYC, RAD21, USP28 and RB1) predicted to change per polysome did not show significant differences at the protein levels (Supplementary Figure S4). Our results show that while loss of MSI2 changes the levels of multiple polysome-associated transcripts, only a subset of those show clear differences in protein levels. Thus, direct binding (as demonstrated by iCLIP peaks) or change in polysome association is not always predictive of changes at the protein level.

### EIF3A is translationally regulated by MSI2

**Figure 6.**
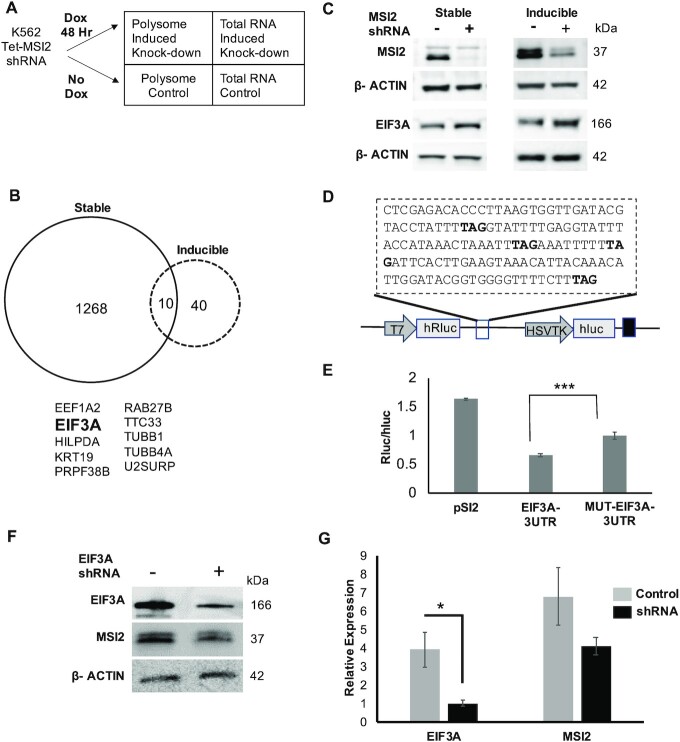
Polysome analysis and luciferase assay highlight EIF3A to be a pronounced MSI2 target. (**A**) Experimental scheme for polysome analysis for MSI2 inducible knockdown (similar to Figure 3C). RNA-seq was performed on paired polysomal RNA and total RNA from induced and uninduced MSI2-KD cells after 48 h of doxycycline induction (in two biological replicates). (**B**) Overlap of the genes from differential analysis of polysome datasets of stable and inducible MSI2-KD cells shows 10 genes to be common in both datasets (stable and inducible knockdown of MSI2). EIF3A was noted to be upregulated in both conditions (2-fold in inducible and 6-fold in stable). (**C**) Western blot showing loss of MSI2 in the stable and inducible MSI2-KD and upregulation of EIF3A levels in both the stable and inducible MSI2-KD along with the loading control (β-actin). EIF3A was upregulated by 1.6 and 1.8-fold (with stable and inducible MSI2 knockdown, respectively) compared to respective controls. (**D**) 3′UTR region of EIF3A transcript cloned in the psiCHECK2 vector downstream of Renilla luciferase (hRluc, driven by an SV40 promoter). The vector also incorporates firefly luciferase (hLuc) driven by an HSVTK promoter as an internal control. TAG highlighted in bold was mutated to TAC to abrogate MSI2 binding. (**E**) Luciferase activity (normalized to firefly luciferase) of various constructs in the psiCHECK2 vector. These vectors were co-transfected with pcDNA-MSI2. These include pSI2 (without any insert), EIF3A 3′UTR and mutant EIF3A 3′UTR. Transfections were performed in NIH3T3 cells and luciferase activity measured 48 h later (*** denote a *P*-value of 0.002). Expression of mutant 3′UTR of EIF3A resulted in increased Renilla to firefly luciferase activity as compared with the unmutated control and the empty vector backbone (control) in NIH3T3 cells. (**F**, **G**) Change to MSI2 from loss of EIF3A. EIF3A knockdown cells were probed for change in MSI2 protein levels by western blotting. EIF3A was reduced 0.2-fold (* denotes a *P*-value of 0.038). MSI2 was noted to reduce by 0.6-fold, but the *P*-value was not significant.

To further distinguish direct targets of MSI2 from indirect ones, we performed polysome profiling of short-term knockdown of MSI2 in a doxycycline-inducible shRNA system. We speculated that direct targets would have an early effect on polysome profiling. We generated single-cell clones of Tet-inducible shRNA directed against MSI2 with reliable inducible knockdown upon doxycycline addition (>80% at 48 h; Figure 6C and Supplementary Figure S5A and B). Polysome profiling was performed as for the stable MSI2 knockdown described earlier (comparing induced and uninduced cells) (Figure 6A and Supplementary Figure S5C and D) (27). A total of 50 genes were shown to change significantly in polysome fraction. We observed that similar to stable knockdown, the change in polysome was more pronounced than for the transcriptome: only MSI2 itself changed at level of transcription, while translation of 50 genes was noted to change (Figure 6B and Supplementary File S1). For the MSI2 inducible knockdown, we also observed that the potential targets of MSI2 were mostly reflected as those transcripts that changed at least 2-fold in polysome profiling and there was no significant change in the total cellular RNA (Supplementary Figure S6A and B). Comparing the datasets (stable versus inducible knockdown), we noted 10 genes that changed in both datasets (Figure 6B). Of these, EIF3A was noted to be upregulated 2-fold in the inducible knockdown and 6-fold in stable knockdown from the polysome profiling results suggesting an immediate and sustained effect from MSI2 knockdown. EIF3A is the largest subunit of eIF3, which plays a central role in the recruitment of the pre-initiation complex to mRNA to initiate peptide translation (45,46). In addition to this role as a canonical regulator of translation, eIF3 components are now recognized to have specialized roles in regulating translation of specific transcripts (47). Other genes in the subset lacked known regulatory functions.

The above findings suggest the possibility that EIF3A is a direct target of MSI2 that may mediate downstream effects of MSI2 overexpression. To test the expectations of this idea, we first confirmed the change in EIF3A expression at the protein level upon MSI2 knockdown in both inducible and stable knockdown cells (Figure 6C); there was no accompanying change in total transcript levels accompanying the changes to total protein (Supplementary Figure S6C and D). Next, we determined whether functionally repressive MSI2 binding to EIF3A 3′UTR transcript could be demonstrated. For this, we cloned the 3′UTR region of the EIF3A transcript within iCLIP peaks into the psiCHECK2 vector downstream of the Renilla luciferase construct (Figure 6D). Mutant 3′UTR (TAC instead of TAG, the minimal MSI2 binding motif) was generated by site-directed mutagenesis. The constructs were co-transfected with MSI2 expression vectors (pcDNA-MSI2) into NIH3T3 cells (chosen given their lack of expression of MSI2) (30). As shown in Figure 6E, expression of mutant 3′UTR resulted in an increase in luciferase activity compared to the wild-type control, suggesting direct binding of MSI2 to the 3′UTR of EIF3A. We also determined reciprocal changes to MSI2 upon loss of EIF3A. As shown in Figure 6F and G, there appears to be a small feedback change in MSI2 protein levels upon EIF3A loss suggesting reciprocal regulation.

**Figure 7.**
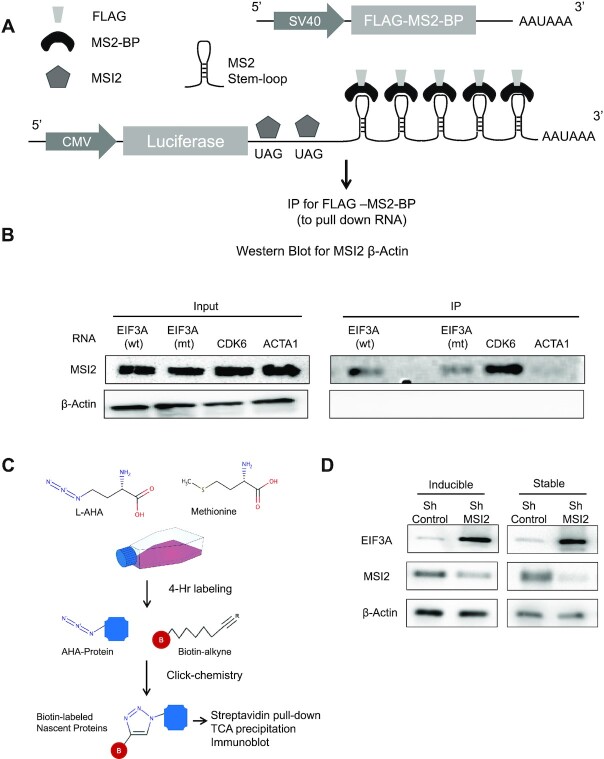
Aptamer pull-down reporter assay and metabolic labeling assay to demonstrate direct MSI2 binding to EIF3A and increased nascent EIF3A protein in response to MSI2 knockdown. (**A**) Scheme of pMS2-LUC-3′UTR vector encoding Renilla luciferase downstream of CMV promoter, 3′UTR, stem loops of MS2 and polyA. Also in the vector is FLAG-MS2-BP expressed from an SV40 promoter. Cell lysates from ∼10–12 million HEK293T cells were immunoprecipitated for FLAG and probed for MSI2 and β-actin control. (**B**) Western blots for MSI2 and β-actin for four constructs with varying 3′UTR [EIF3A wild type (wt), EIF3A mutant (mt), CDK6 and ACTA1]. EIF3A mutant construct had TAG mutated to TAC. Input cell lysates as well as IP eluates were probed separately to determine relative enrichment. (**C**) Scheme for metabolic labeling and immunoblotting for nascent EIF3A protein. l-AHA is a methionine analogue added to cell cultures for 4 h. l-AHA-labeled proteins are then biotinylated with click chemistry enabling their isolation with streptavidin beads, precipitation with TCA and immunoblotting. (**D**) Immunoblotting for nascent EIFA protein following labeling with l-AHA and click chemistry. Acute depletion of MSI2 through inducible shRNA as well as stable MSI2-KD cells is shown along with controls. Precipitated proteins after streptavidin pull down were assayed for EIF3A, MSI2 and β-actin.

To directly determine whether EIF3A mRNA recruits stably bound MSI2 *in vivo*, we developed an orthogonal assay. Specifically, an aptamer pull-down assay was designed whereby five stem loops from the bacteriophage MS2 were cloned into several different 3′ UTRs (pMS2-LUC-3′UTR) placed downstream of an open reading frame encoding luciferase and constitutively expressed (Figure 7A). The 3′UTRs tested were wild-type EIF3A 3′UTR, mutant EIF3A (with mutated UAG), and positive and negative controls (3′UTR of CDK6 and ACTA1 mRNAs, respectively). A FLAG-tagged MS2 binding protein (FLAG-MS2-BP) was co-expressed to enable independent pull down of each expressed reporter mRNA from cell lysates: immunoprecipitates of FLAG-MS2-BP were subjected to western blotting and probed for MSI2 and β-actin with specific antibodies. Figure 7B shows that MSI2 is enriched in the IP from lysates expressing wild-type EIF3A 3′UTR and positive control (CDK6 3′UTR), but not in negative control (ACTA1 3′UTR). Importantly, mutating the UAG sites in the positive control results in reduction of MSI2 confirming loss of binding to mutant EIF3A 3′UTR. We also performed western blot analysis of cell lysates for luciferase expression in the pull-down samples (Supplementary Figure S7A), which confirmed increase in the EIF3A mutant with respect to wild-type control. Transcript levels of luciferase did not show a similar change (Supplementary Figure S7B and C). Together, we conclude that MSI2 binds the EIF3A 3′UTR strongly and specifically *in vivo*.

Finally, to demonstrate direct translational repression of EIF3A by MSI2, we determined changes to nascent protein levels by metabolic labeling (Figure 7C). Cells in methionine-free media were labeled with l-AHA, a methionine analogue, for 4 h followed by click chemistry with biotin alkyne to biotinylate the labeled proteins. Protein was precipitated and biotin-labeled fraction pulled down with streptavidin beads and analyzed for EIF3A, MSI2 and β-actin. As shown in Figure 7D, for MSI2-KD clones (both stable and inducible loss of MSI2), EIF3A nascent peptide translation was noted to be higher after 4 h of labeling compared to control clones. These results show that change in protein levels of EIF3A upon MSI2 loss arises from an actual increase in peptide translation.

**Figure 8.**
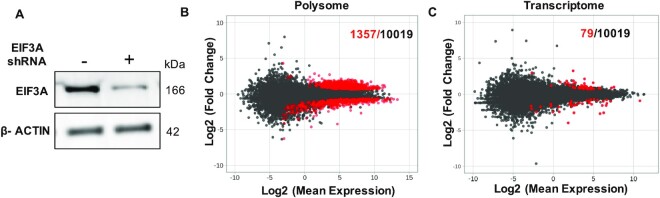
Polysome and transcriptome analyses after EIF3A inducible knockdown. (**A**) Western blot of uninduced and induced EIF3A knockdown cells after 72 h of doxycycline treatment shows significant loss of EIF3A levels with EIF3A shRNA. (**B**) MA plot (log_2_ mean expression plotted against log_2_ mean fold change) for polysome profiling of uninduced and induced EIF3A knockdown after 72 h. Red dots indicate the transcripts that changed significantly and black dots represent the total number of transcripts identified. (**C**) MA plot (log_2_ mean expression plotted against log_2_ mean fold change) for transcriptome from polysome profiling of uninduced and induced EIF3A knockdown after 72 h. Red dots indicate the transcripts that changed significantly and black dots represent the total number of transcripts identified.

A final expectation of our working model that EIF3A is a direct target of MSI2 is that increased MSI2 will lead to downstream changes in translation. To address this, we reasoned that reduction in EIF3A concentration in cells should phenocopy at least some of the effects of MSI2 overexpression. Thus, doxycycline-inducible knockdown cells of EIF3A were generated using lentiviral vectors expressing two independent shRNAs. We first verified knockdown of EIF3A upon doxycycline induction (for both transcript and protein; Figure 8A and Supplementary Figure S8A–C). Accordingly, we selected 72 h of induction as the optimum duration of doxycycline induction and performed polysome profiling of EIF3A knockdown cells. We found that similar to MSI2, knockdown of EIF3A predominantly affects the polysome (1357 transcripts) (Figure 8B) compared to total transcripts (79 transcripts) (Figure 8C and Supplementary File S3). Finally, to determine how acute loss of EIF3A changes in polysome-associated transcripts compared to those induced by MSI2 loss, we compared the two datasets (transcripts decreased in polysomes upon EIF3A loss compared to those that increased upon stable MSI2 knockdown). As shown in Supplementary Figure S9A and B, seven transcripts overlapped. This surprisingly limited correlation in the transcriptome-wide analyses could represent the most robust hits, given our multiple independent biochemical approaches showing direct MSI2 binding to the EIF3A 3′UTR and reduction of EIF3A protein levels. We hence conclude that EIF3A is an early, direct target of MSI2.

## DISCUSSION

In this study, we have explored the molecular mechanisms that underlie translational repression by MSI2 in myeloid leukemia cells. Upregulation of both Musashi homologues MSI1 and MSI2 has been reported in numerous neoplasms, including aggressive myeloid leukemia (48). Previous studies have used CLIP and SELEX to define sequence specificity of Musashi binding (16,19,35–37,49) and these studies generally agreed on two important findings: (i) several hundreds to thousands of targets are bound by Musashi proteins (16,35) and (ii) binding regions are enriched for the trinucleotide motif UAG (19,37). These results are in contrast with previous reports that focused on few select targets of functional significance, such as the Notch inhibitor NUMB and cyclin-dependent kinase inhibitor p21^Cip1^ (36,50). Binding of MSI1 and MSI2 to thousands of targets enriched by the UAG motif was surprising given their relatively narrow physiological role to regulate asymmetric cell division and quiescence. Here, we integrated two genome-wide approaches (iCLIP and polysome profiling) to test the hypothesis and identify specific functional targets of MSI2. Our iCLIP analysis revealed over 4000 high-confidence RNA binding partners of MSI2 in K562 cells with enrichment for UAG- and polyU-containing pentamers, in agreement with previous results (14–16). In contrast, polysome analysis of cells with stable knockdown of MSI2 showed that discernible changes at the level of translation are seen only in a small fraction (2.3%) of the transcripts bound by MSI2 per iCLIP. Additionally, changes in translation were more pronounced than those in the transcriptome (only 0.39%), confirming the primary role of MSI2 as a translational regulator.

Previous studies that employed the related technique ribosome profiling noted low coverage of ribosome footprints to be a limitation that likely underestimated translational changes (37,49). Given that our experiments were performed in stable knockdown cells, the polysome dataset likely contains both direct and indirect targets. We presumed that direct targets of MSI2 are expected to have iCLIP peaks associated; accordingly, 54% of transcripts were translationally upregulated. In comparison, 26% of transcripts that were downregulated in response to MSI2 knockdown had iCLIP peaks. Interestingly, about a quarter of all transcripts detected in the polysome fraction had significant iCLIP peaks. Thus, a mere change in translation after stable MSI2 knockdown cannot be interpreted as a direct effect of MSI2. The absence of iCLIP peaks in transcripts with discernible change in translation likely points to an effect of downstream mediators regulated by MSI2. It is important to note that while both Musashi proteins are thought to be a translational repressor, it can serve as a translational activator in certain cellular contexts (51). This further complicates inferring direct or indirect functional effects of MSI2 binding.

Through further in-depth analyses of the iCLIP and polysome datasets, we were able to define some parameters that distinguish RNA targets with productive binding from those bound nonproductively. Productive targets had higher UAG content of cross-link clusters as well as higher total number of clusters and cluster density within the transcript coordinates. We suspect that our analysis was somewhat constrained by the presence of both direct and indirect targets within these datasets, because about a quarter of all polysome-associated transcripts had high-confidence cross-link clusters, while slightly more than half of the upregulated transcripts have similar iCLIP clusters. The wide variation of UAG content and cluster density suggests that other factors, such as additional proteins or a specific cell context, may modulate targeting. Despite this limitation, the highly significant differences between the subsets support the notion that productive binding of MSI2 likely involves multiple MSI2 binding events on each affected target.

To definitively identify direct targets of MSI2, we performed polysome profiling after acute depletion of MSI2 (through doxycycline-inducible shRNA). Notably, far fewer transcripts (50) were noted to change on acute MSI2 loss. Of these, we chose to pursue EIF3A given it was one of the few transcripts with sustained upregulation upon stable MSI2 knockdown. In addition to its role in canonical initiation of translation, EIF3 is also now known to regulate specific transcripts that regulate cell proliferation (47). Using multiple complementary approaches, we show that MSI2 is not only bound to EIF3A, but the binding regulates translation of nascent protein. While the role of the EIF3 complex in translation initiation is well known, its own regulation by translational repressors such as MSI2 is novel. Multiple components of the EIF3 are now known to be dysregulated in multiple cancers (EIF3A in breast, cervical, lung and gastric cancers; EIF3B in breast and gastric cancers; EIF3C in testicular cancers; and EIF3H in prostate cancers) (52). It is currently unclear whether these changes drive oncogenesis or reflect altered protein translation in response to cellular growth. Given that Musashi proteins broadly regulate symmetry of cell division and stem cell quiescence, it is likely that the effect on EIF3 may be broadly relevant across developmental stages and merits further investigation.

Finally, we performed polysome profiling of EIF3A knockdown cells to determine intersection of MSI2 and EIF3A targets. Like MSI2, EIF3A was also noted to predominantly change in polysome-associated mRNA as compared to total mRNA levels. These transcripts did not, however, correlate with those changed by MSI2. We speculate several potential reasons: (i) EIF3A is a critical component of the canonical EIF3 complex and its acute loss may have complex, pleiotropic effects not discernible by polysome profiling even with acute loss. (ii) Given that MSI2 loss results in increase in EIF3A, an overexpression model of EIF3A may be more physiological to study the intersection of its targets with MSI2. Thus, despite strong, biochemical data showing functional perturbation of EIF3A by MSI2, downstream effects of MSI2 are likely complex and not attributable to a single mediator. Our studies primarily employed shRNA-based knockdown; it is conceivable that overexpression studies may have revealed additional changes to polysome occupancy. While our results show strong biochemical evidence of EIF3A regulation by MSI2, we acknowledge that the relevance of such a regulation in cancer can only be inferred, and hence a limitation of the study. CDK6 was another target noted to be bound by MSI2 with demonstrable changes to protein levels and 3′UTR binding. Given both CDK6 and MSI2 have oncogenic roles (53), CDK6 upregulation upon loss of MSI2 is surprising, but underscores the overall pleiotropic effects of MSI2 on the proteome.

Finally, our results also highlight overall challenges in determining functional targets of RBPs. Cross-link IP has helped define the RNA targets bound by dozens of RBPs, but mere binding is not synonymous with a discernible functional change. Computational algorithms that rely on intensities and positions of binding peaks have been proposed to distinguish functional binding (54). Our own results also show that functional targets of MSI2 binding correlate with some metrics of higher density binding in iCLIP data. Polysome profiling pointed to some targets such as EIF3A; other targets were not picked up on immunoblotting. It is unclear whether additional approaches to define translatome (such as ribosome profiling) will provide higher specificity to this end. We conclude that these limitations are to be kept in mind while using transcriptome-wide approaches to define functional targets of RBPs, such as MSI2.

## DATA AVAILABILITY

Sequencing files have been deposited in Gene Expression Omnibus (GSE93210). Files from iCLIP analysis are available at https://github.com/pillailab/MSI2.

## Supplementary Material

zcac015_Supplemental_Files
