# Supplementary material for: Integrative genome-wide analysis reveals EIF3A as a key downstream regulator of translational repressor protein Musashi 2 (MSI2)
Source: NAR Cancer. 2022 May 2;4(2):zcac015. doi: 10.1093/narcan/zcac015 (PMC9070473; doi:10.1093/narcan/zcac015)
Supplement: zcac015_Supplemental_Files [file zcac015_supplemental_files.zip › SUPPLEMENTARY FIGURES_R1_03142022.pptx]

## Slide 1
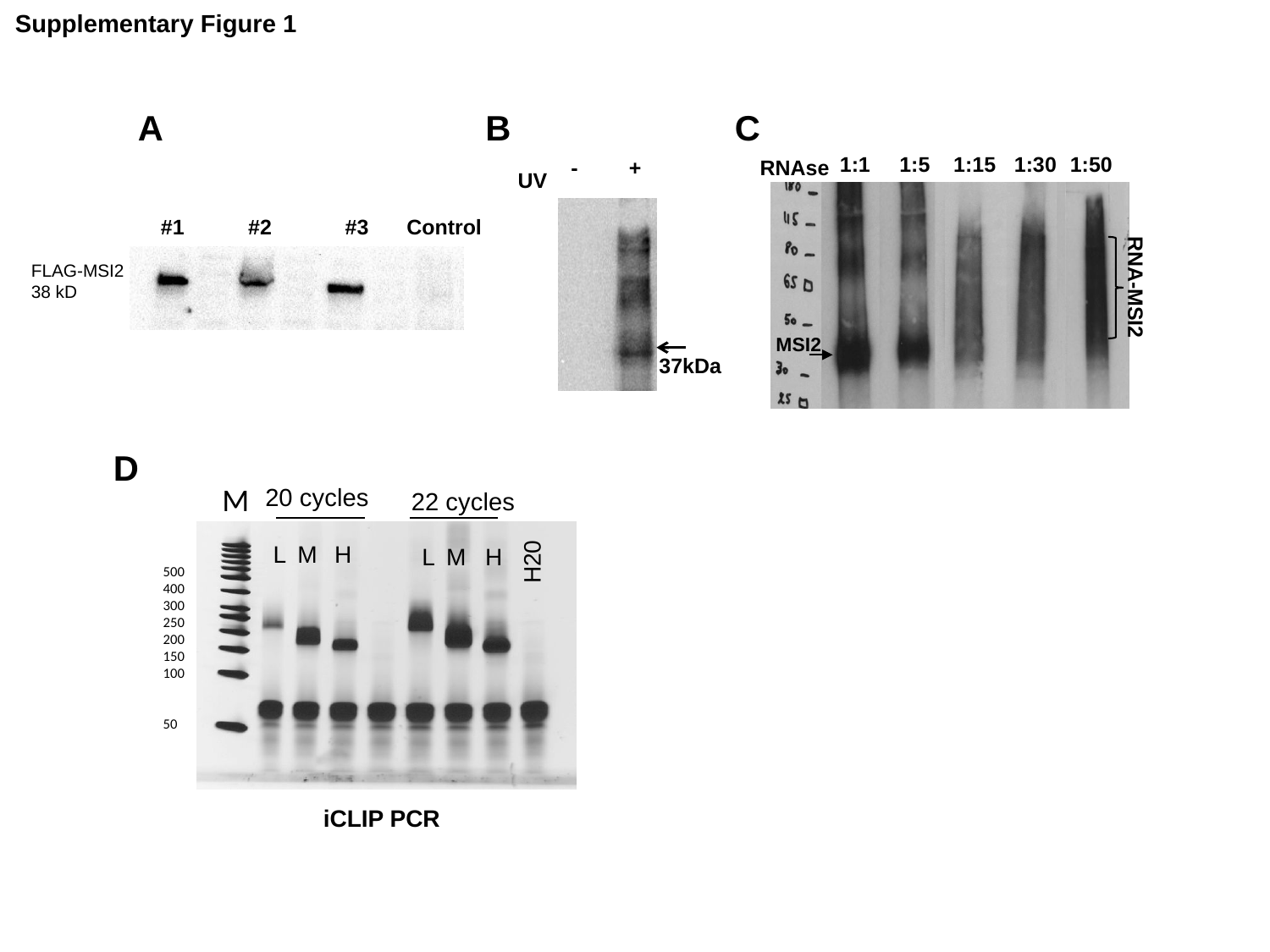

Supplementary Figure 1
 A
B
C
1:1
1:5
1:15
1:30
1:50
RNAse
RNA-MSI2
MSI2
-
+
UV
#1
#2
#3
Control
FLAG-MSI2
38 kD
37kDa
D
M
20 cycles
22 cycles
L
M
H
L
M
H
H20
iCLIP PCR
500
400
300
250
200
150
100
50

## Slide 2
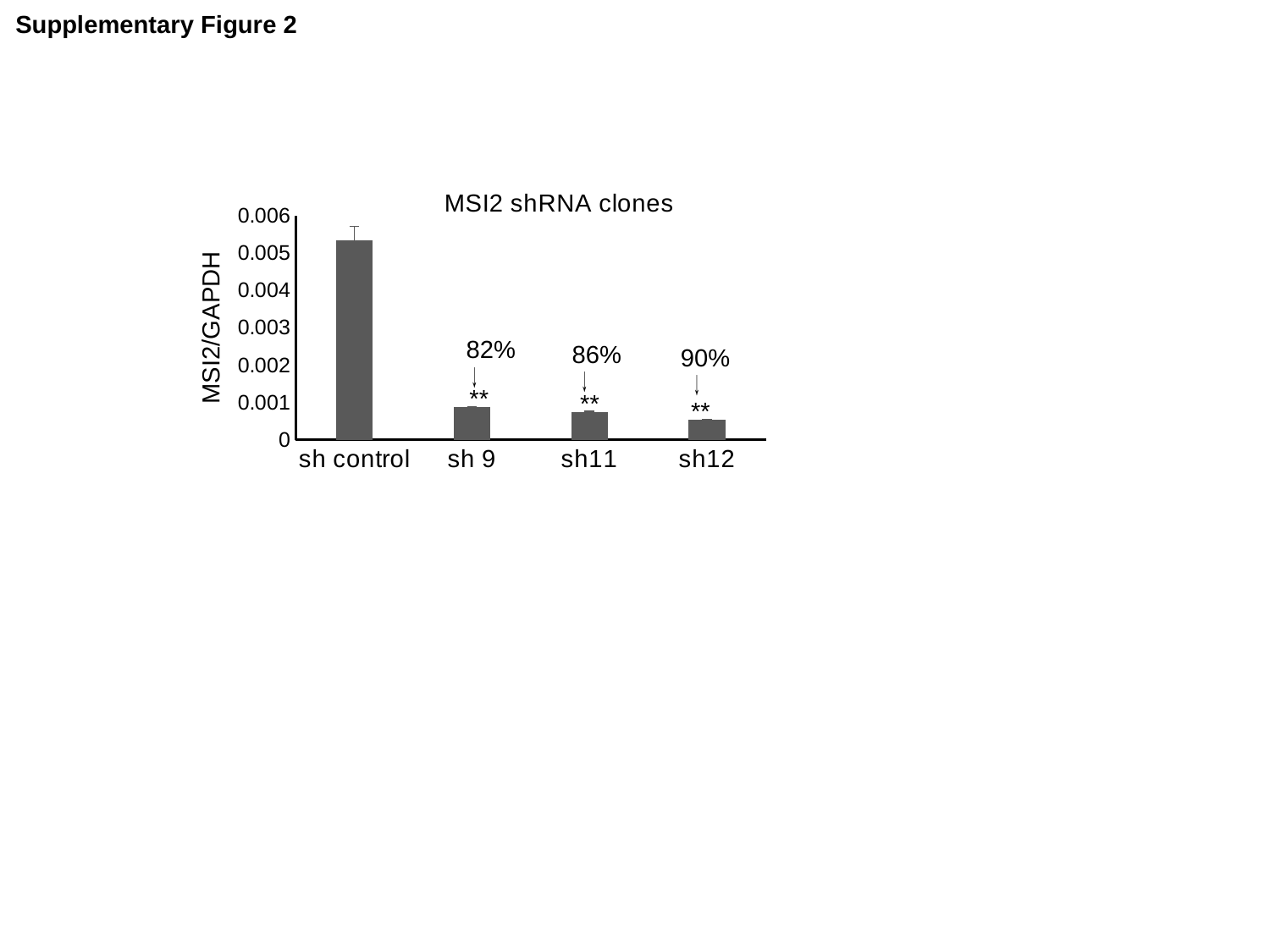

Supplementary Figure 2
### Chart: MSI2 shRNA clones
| Category | |
|---|---|
| sh control | 0.00533192927461472 |
| sh 9 | 0.000881398025242591 |
| sh11 | 0.000731157415126469 |
| sh12 | 0.000540111530552364 |MSI2/GAPDH
82%
86%
90%
**
**
**

## Slide 3
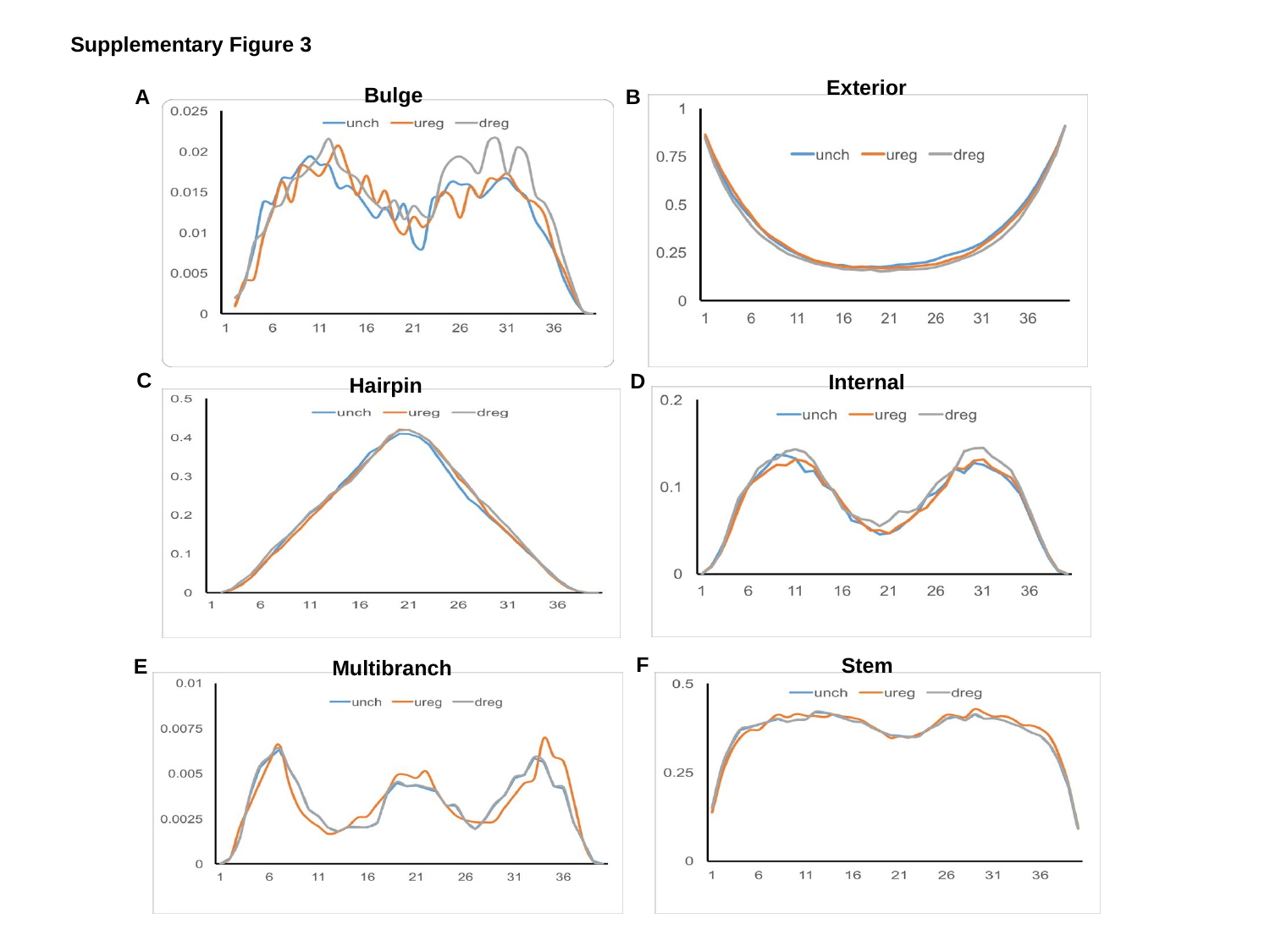

Supplementary Figure 3
Exterior
Bulge
A
B
C
D
Internal
Hairpin
F
Stem
E
Multibranch

## Slide 4
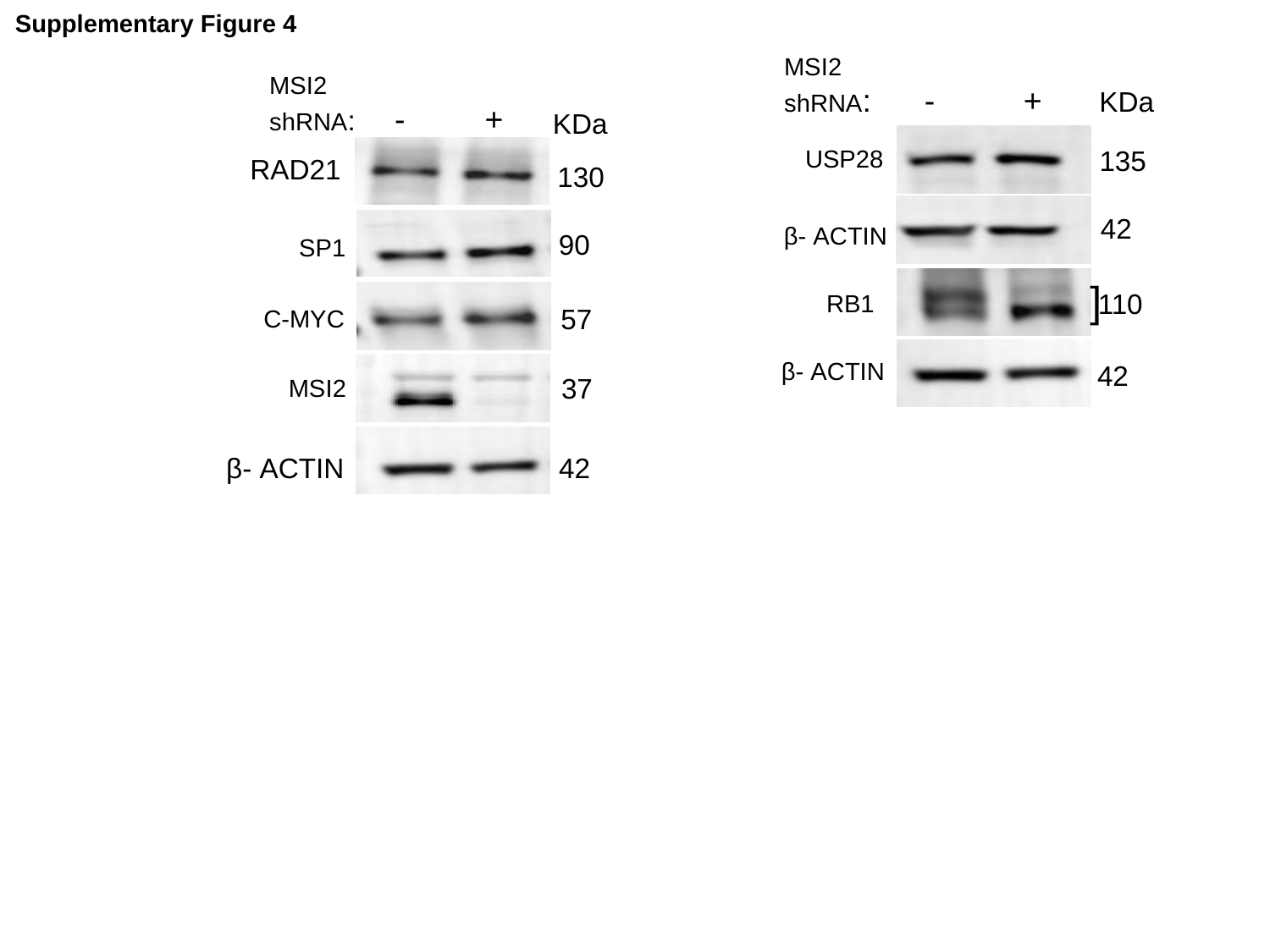

Supplementary Figure 4
MSI2
shRNA: - +
MSI2
shRNA: - +
KDa
KDa
135
USP28
RAD21
130
42
90
SP1
β- ACTIN
]
 RB1
110
57
C-MYC
β- ACTIN
42
37
MSI2
42
β- ACTIN

## Slide 5
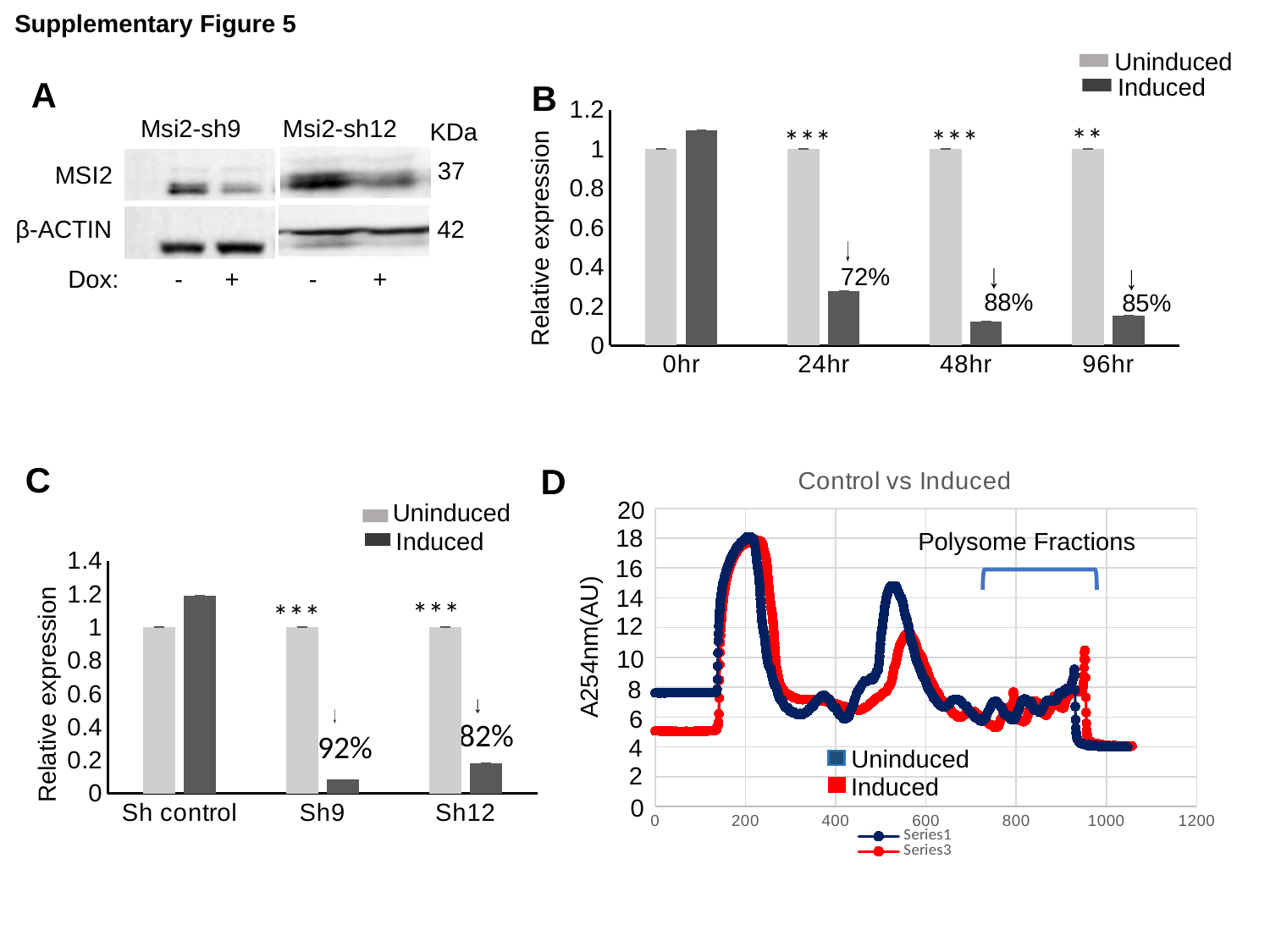

Supplementary Figure 5
Uninduced
**
***
***
Relative expression
Induced
A
B
Msi2-sh9 Msi2-sh12
KDa
37
MSI2
β-ACTIN
42
72%
Dox: - + - +
88%
85%
### Chart: Control vs Induced
| Category | | |
|---|---|---|C
***
***
Relative expression
82%
92%
D
20
Uninduced
18
Polysome Fractions
Induced
16
14
12
A254nm(AU)
10
8
6
4
Uninduced
Induced
2
0

## Slide 6
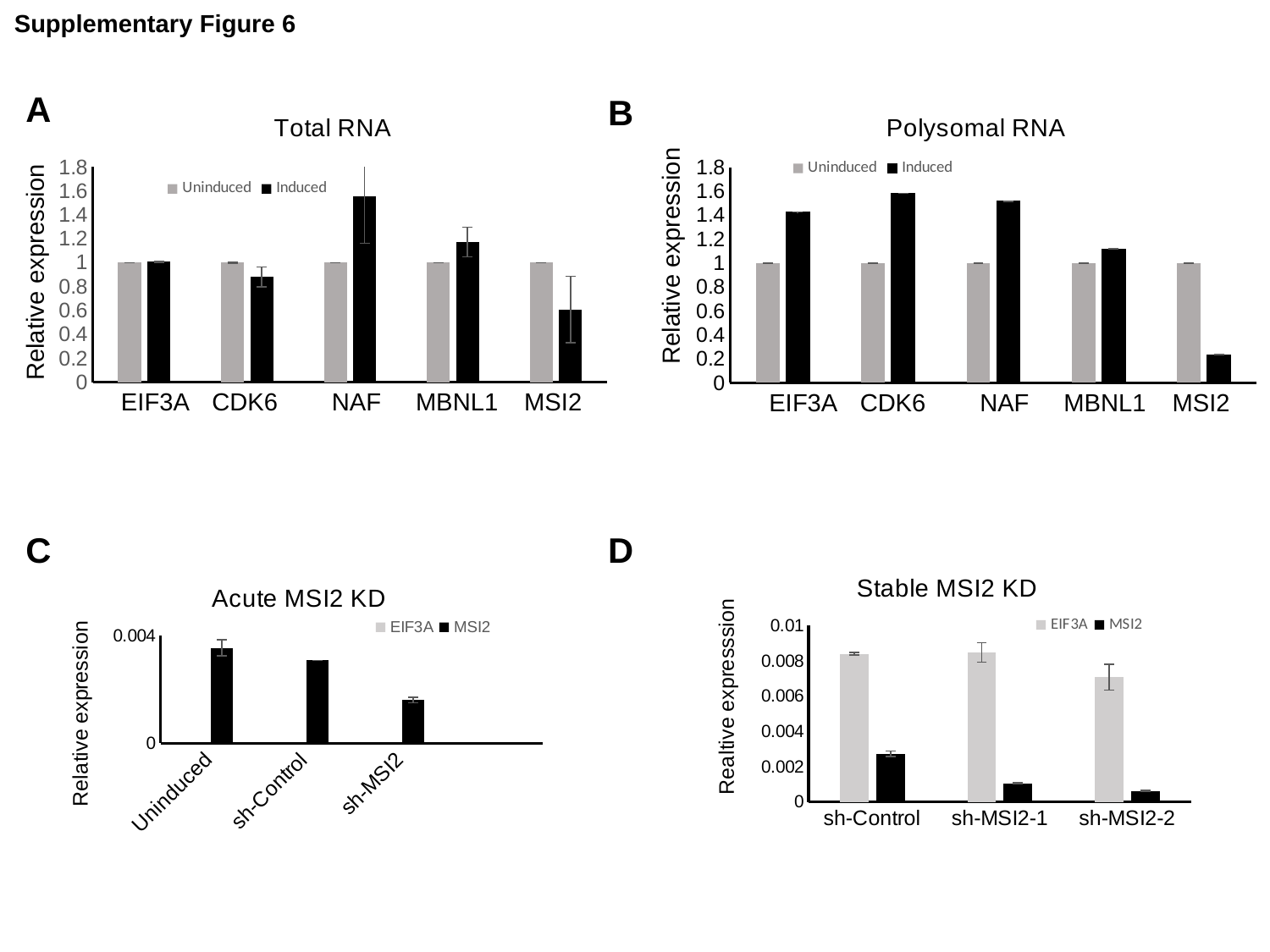

Supplementary Figure 6
A
B
### Chart: Total RNA
| Category | Uninduced | Induced |
|---|---|---|
| EIF3A | 1.0 | 1.0087573585624814 |
| CDK6 | 1.0 | 0.8815135928934298 |
| NAFT | 1.0 | 1.555430068994563 |
| MBLN | 1.0 | 1.1739764163487587 |
| MSI2 | 1.0 | 0.6080353950836302 |
### Chart: Polysomal RNA
| Category | Uninduced | Induced |
|---|---|---|
| EIF3A | 1.0 | 1.42721524239741 |
| CDK6 | 1.0 | 1.5834326671237677 |
| NAFT | 1.0 | 1.5164714250833728 |
| MBNL1 | 1.0 | 1.119961334745309 |
| MSI | 1.0 | 0.23571666348441206 |Relative expression
Relative expression
EIF3A
CDK6
NAF
MBNL1
MSI2
EIF3A
CDK6
NAF
MBNL1
MSI2
C
D
### Chart: Stable MSI2 KD
| Category | EIF3A | MSI2 |
|---|---|---|
| sh-Control | 0.00841203584459218 | 0.002736735828381004 |
| sh-MSI2-1 | 0.008486128284308902 | 0.001064495195739161 |
| sh-MSI2-2 | 0.007088630235451568 | 0.0006184575618891988 |
### Chart: Acute MSI2 KD
| Category | EIF3A | MSI2 |
|---|---|---|
| Uninduced | 0.008909335502121887 | 0.0035538270292405794 |
| sh-Control | 0.010879436792435727 | 0.0030834990661352366 |
| sh-MSI2 | 0.010443093941332231 | 0.0016073417049190138 |

## Slide 7
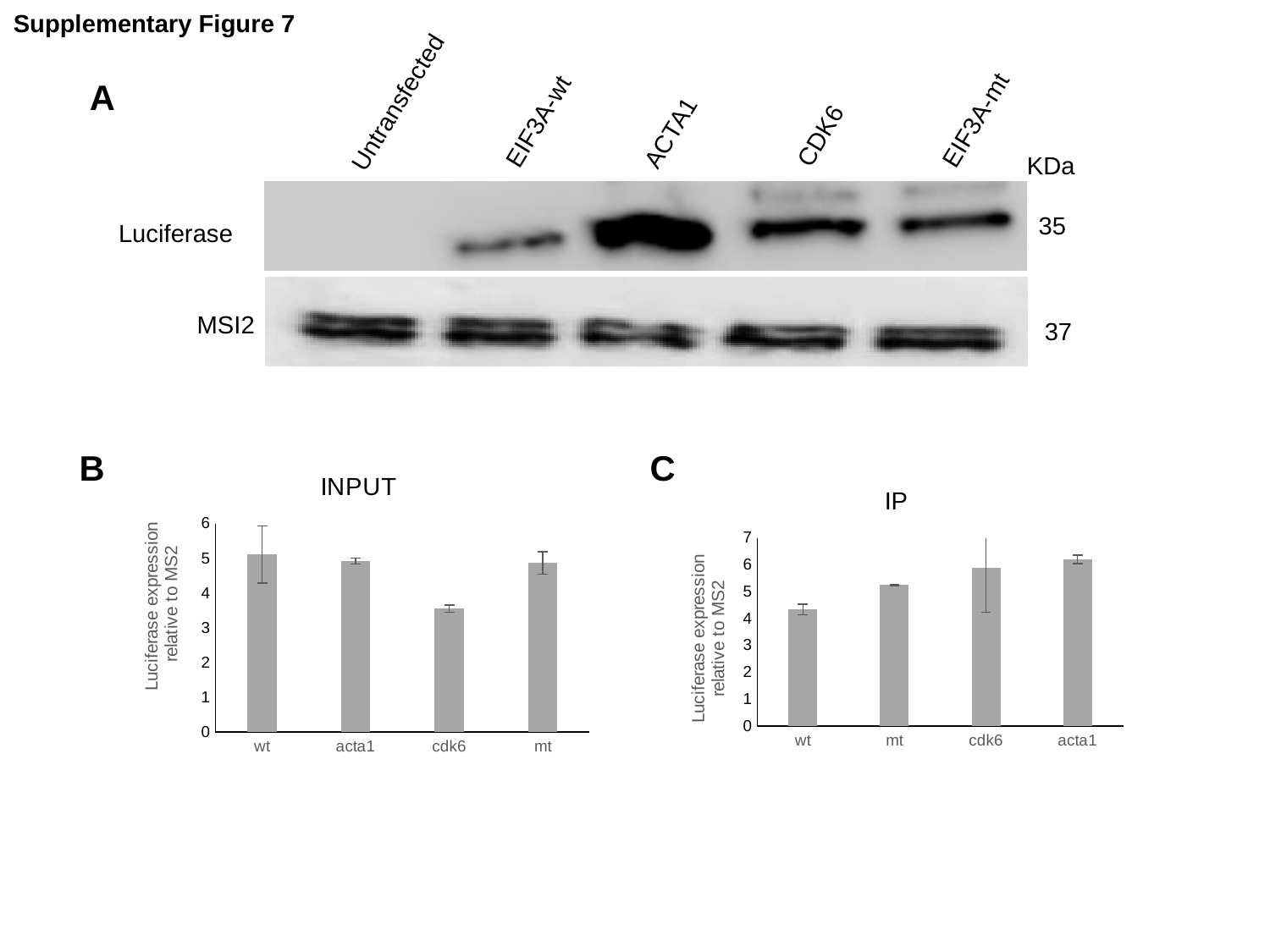

Supplementary Figure 7
A
ACTA1
Untransfected
EIF3A-mt
EIF3A-wt
CDK6
KDa
 35
Luciferase
MSI2
37
B
C
### Chart: INPUT
| Category | |
|---|---|
| wt | 5.107727560458188 |
| acta1 | 4.919741647066174 |
| cdk6 | 3.551410054059443 |
| mt | 4.8638581635430835 |
### Chart: IP
| Category | |
|---|---|
| wt | 4.327086930499011 |
| mt | 5.244711137875367 |
| cdk6 | 5.869470428121842 |
| acta1 | 6.2038535460553375 |

## Slide 8
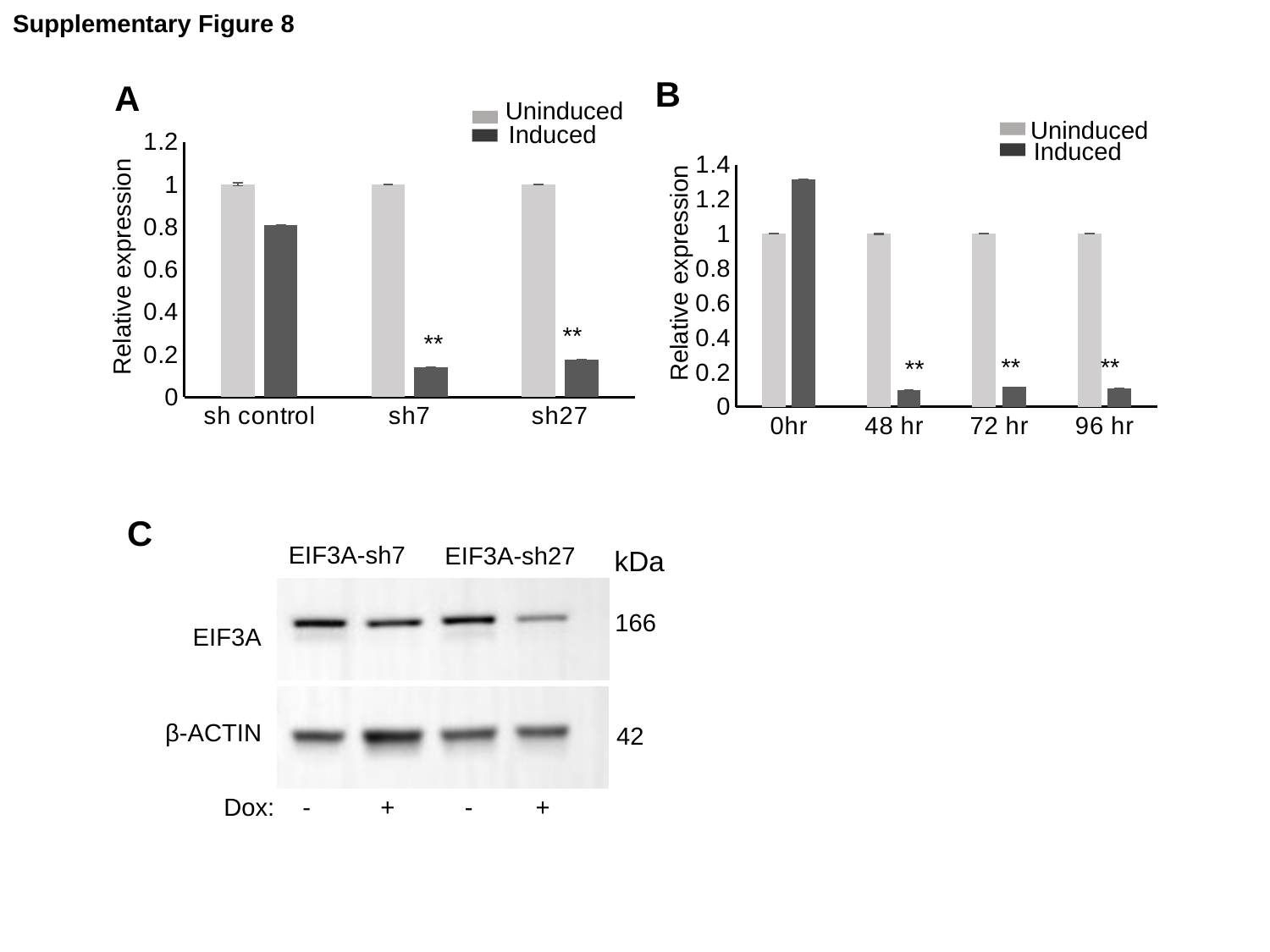

Supplementary Figure 8
B
A
Uninduced
Uninduced
Induced
### Chart
| Category | Control | Dox |
|---|---|---|
| sh control | 1.0 | 0.806942840168894 |
| sh7 | 1.0 | 0.140793059766377 |
| sh27 | 1.0 | 0.176141787649929 |
Induced
### Chart
| Category | Control | dox |
|---|---|---|
| 0hr | 1.0 | 1.314035338548172 |
| 48 hr | 1.0 | 0.0966208034099151 |
| 72 hr | 1.0 | 0.113950918189668 |
| 96 hr | 1.0 | 0.105355558776756 |Relative expression
Relative expression
**
**
**
**
**
C
EIF3A-sh7
EIF3A-sh27
EIF3A
β-ACTIN
Dox: - + - +
kDa
166
42

## Slide 9
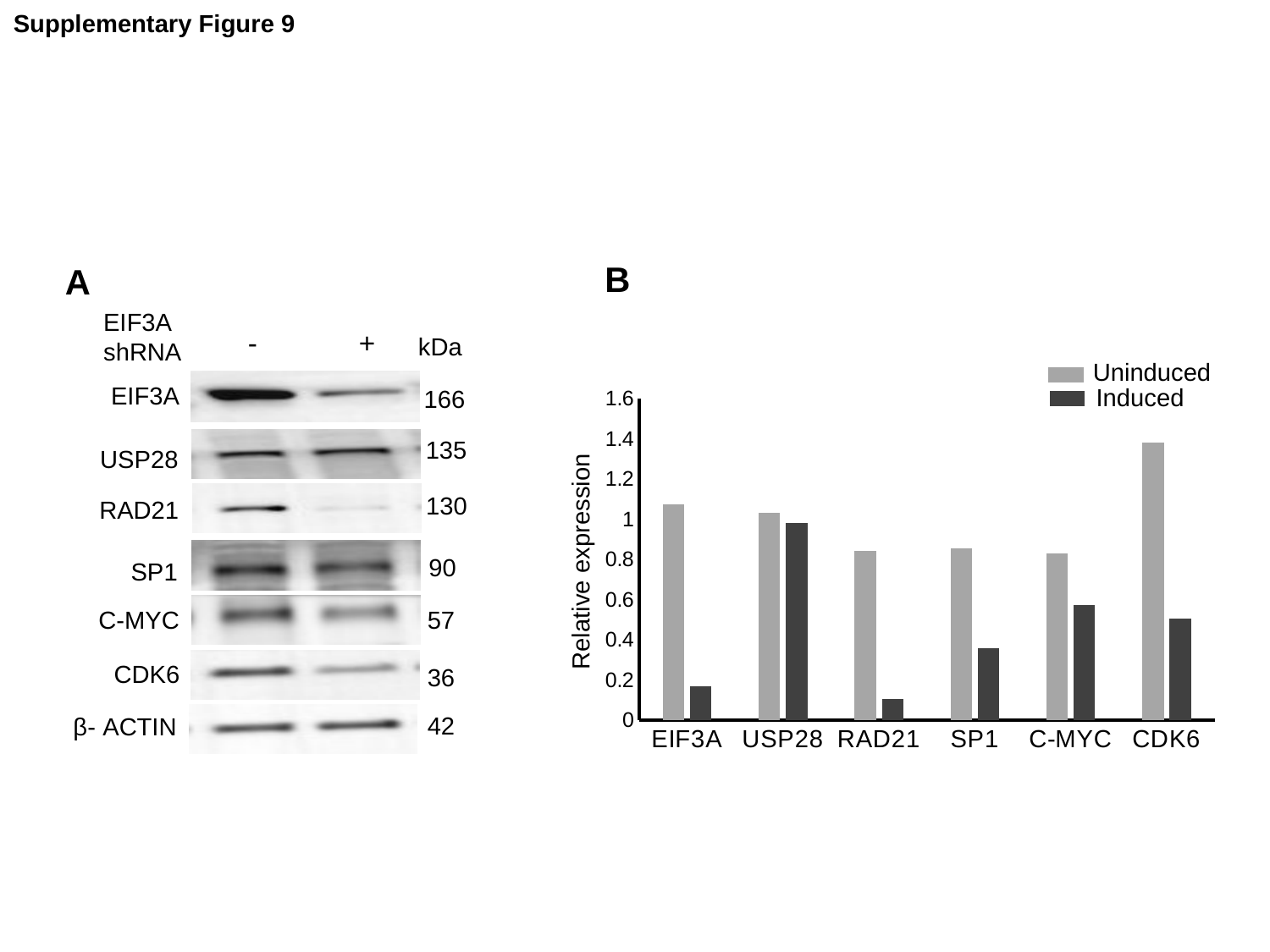

Supplementary Figure 9
B
Uninduced
Induced
### Chart
| Category | Uninduced | Induced |
|---|---|---|
| EIF3A | 1.0724509396096187 | 0.16884437571721933 |
| USP28 | 1.0302832745879849 | 0.9818698248758807 |
| RAD21 | 0.840771606655842 | 0.10522270313905835 |
| SP1 | 0.8553333718754378 | 0.359222014024113 |
| C-MYC | 0.828675579614526 | 0.5723464474450348 |
| CDK6 | 1.3825163983090336 | 0.5065504221520051 |
Relative expression
A
EIF3A
shRNA
 - +
kDa
EIF3A
166
135
USP28
130
RAD21
90
SP1
C-MYC
57
CDK6
36
42
β- ACTIN
